# On the Parental Influence on Children’s Physical Activities and Mental Health During the COVID-19 Pandemic

**DOI:** 10.3389/fpsyg.2022.675529

**Published:** 2022-03-25

**Authors:** Fatemeh Khozaei, Claus-Christian Carbon

**Affiliations:** ^1^Department of Architectural Engineering, Dhofar University, Salalah, Oman; ^2^Ergonomics, Psychological Aesthetics, Gestalt, Bamberg, Germany; ^3^Department of General Psychology and Methodology, University of Bamberg, Bamberg, Germany

**Keywords:** COVID-19, children mental health, mental health, parental stress, physical activity, perceived risk, physical activity avoidance

## Abstract

**Background:**

While neighborhood safety and stranger danger have been mostly canonized to play a part in parents’ physical activity (PA) avoidance, less is known about the impact of parental stress and perceived risk on children’s PA avoidance and consequently on children’s level of PA and wellbeing. Understanding the contributors to children’s wellbeing during pandemic disease is the first critical step in contributing to children’s health during epidemic diseases.

**Methods:**

This study employed 276 healthy children, aged 10–12 years, and their parents. Data were collected in October and November 2020, about 9 months after the local closing of schools due to the coronavirus disease 2019 (COVID-19) pandemic. Parents and children answered a separate set of questions. Besides the demographic information, the parents responded to questions on their stress level, perceived risk of COVID-19, and PA avoidance for children. Children responded to questions on their PA and wellbeing in the last week. Data were analyzed using SmartPLS and IBM SPSS 22.

**Results:**

The result of the study supported the four directional research hypotheses of the sequential study model. As hypothesized, parents’ stress and perceived risk levels of COVID-19 negatively affected children’s PA. The PA level was shown to predict children’s wellbeing and mental health. Housing type, parents’ job security, number of siblings, number of members living together in-home, and history of death or hospitalization of relatives or family members due to COVID-19 were found to be associated with parents’ stress and children’s mental health.

**Conclusion:**

This study sheds light on parents’ role in children’s wellbeing and mental health during the COVID-19 pandemic. Parents with higher stress and high restrictive behaviors might put their children at risk of mental disorders in the end.

## Introduction

Many mental health disorders among adults find their roots in early childhood (Thomson et al., 2019). Studies suggest that mental disorders can affect children’s various aspects of life, such as educational outcomes (Patel et al., 2007), attentional function (Vloet et al., 2010), and eating behavior (Hill et al., 2018). Accordingly, understanding factors contributing to children’s mental health is vitally important for reducing the risk of mental disorders later in adulthood. Physical activity (PA) has been emphasized repeatedly among various factors that predict children’s mental health. Previous studies have covered various benefits of PA on children’s life (Christiansen et al., 2018; García-Hermoso et al., 2020a). Studies suggest that PA affects children’s health, quality of life (Shoesmith et al., 2020), and sleep quality (Chong et al., 2020). The long-term PA is an essential measure in balancing blood pressure, insulin level, and wellbeing (Leary et al., 2008).

Being aware of its benefits has not guaranteed a high level of PA among children of various ages globally. In studying children’s PA, there has been an agreement that compared to the past, children’s active transportation participation, such as walking and cycling to school, has decreased significantly (Salmon et al., 2005). Despite the proven benefits of active plays in children’s PA level (Chong et al., 2020; García-Hermoso et al., 2020a; Shoesmith et al., 2020), most children in present times show lower outdoor playing activities than previous generations (Valentine and McKendrck, 1997; Hillman, 2006). According to WHO, children and adolescents aged 5–17 years should at least do 1 h of moderate-to-vigorous intensity activities on average.

Children might do not get engaged in recommended weekly PA regularly (Cavill et al., 2001). The level of children’s PA is associated with various factors. Previous studies suggest that children’s disabilities (Bedell et al., 2013), desire and motivation for physical activities (Wohlfarth et al., 2013), gender (Loucaides and Jago, 2008), and self-perception (Wright et al., 2019) can make a difference in the PA level of children in comparison with their counterparts.

Parents also might prevent or encourage children’s PA in various ways and for multiple reasons. For example, children’s independent mobility (CIM) is considered an essential source of PA (Carver et al., 2012). CIM is defined as children’s freedom and ability to move around public spaces without any adults’ absence or supervision. It covers activities such as walking, cycling, or independent playing. Studies suggest that CIM positively affects children’s PA and weight status (Schoeppe et al., 2013).

Meanwhile, CIM is mainly dependent on parents’ permission. Parental fear and concern about traffic safety and strangers are associated with more constraints on CIM (Carver et al., 2010). Parents’ decisions substantially determine the extent and range of independence mobility (Carver et al., 2010). Compared to the past, nowadays, parents do restrict children much more from playing in local parks or streets (Karsten, 2005).

More recently, the coronavirus disease 2019 (COVID-19) pandemic has been an additional reason for reducing children’s PA. COVID-19 added to the complexity of managing children’s PA. The closing of schools plus online education eliminated school-based PA from children’s life. Before COVID-19, children might spend 5–40% of recommended PA time at school (Ridgers et al., 2006). Besides, parents are aware of the benefits of PA for children; they are also mindful of the negative consequences of a lack of PA on overweight or even obesity (Towns and D’Auria, 2009; Norman Ã. et al., 2015). Accordingly, the decision-making and managing of children’s PA during the COVID-19 pandemic are much more on the parents’ side than before the pandemic (at least as long as there are no general curfews on all possible PA modes, of course).

Due to the importance of PA and its demonstrated positive effects on children, the issue is well-covered in the literature, yet less is known about the factors that the parental PA restrictions impact children’s perceived wellbeing and mental health during the COVID-19 pandemic. Addressing the impact of the parental decision on PA level of children and their perceived consequences of these decisions provides a piece of valuable information that hardly could be examined in studies that were confined to the pre-COVID-19 era (Timperio et al., 2004; Weir et al., 2006; Schoeppe et al., 2013; Wohlfarth et al., 2013). This study attempts to shed light on the perceived impact of PA level on children’s wellbeing and mental health during the COVID-19 pandemic. Such an evaluation has to address the system of parents-children, so we included the perspective of the impact of COVID-19 on parents to gain a complete picture. Such effects can be classified regarding psychological states such as increased stress level (Brown et al., 2020; Xu et al., 2020), anxiety (Drouin et al., 2020), mental health (Limbers et al., 2020; Wu et al., 2020), as well as physical and wellbeing states (Patrick et al., 2020).

The result of this study can fill a part of the gap in previous studies. First, most of our knowledge of factors contributing to children’s wellbeing is limited to the pre-COVID-19 pandemic. Second, most studies on parents’ PA avoidance factors have emphasized neighborhood safety and stranger danger. Less is known about the impact of parents’ stress and perceived risk in this regard. By addressing these issues, we attempted to contribute to studying children’s mental health during epidemic diseases. Keeping in mind the effect of mental disorders on other aspects of children’s lives and even its consequences on their adult mental health, it is essential to study factors contributing to children’s wellbeing and mental health. This study presents pioneering research on parents’ role in children’s wellbeing and mental health during the COVID-19 pandemic.

## Theoretical Framework

Before COVID-19, mental health was a central and critical topic for research: it is estimated that more than 10% of children and adolescents around the world have a mental disorder (Kato et al., 2015), particularly in a pandemic crisis of this extent such figures and further potential deterioration of this condition, mental disorders have to be focused. People were affected concerning wellbeing and mental health, not only adults (Yeasmin et al., 2020) but also children (Parola et al., 2020). Among various factors contributing to children’s mental health, we concentrated on the role of PA. We focused on the parental role in reducing children’s PA during the COVID-19 pandemic and its consequences on children’s mental health and wellbeing. We attempted to canonize the role of parents in PA avoidance.

The literature well covers parents’ influence on children’s PA (Sallis et al., 2000). Stress and perceived risk have been shown to affect parents’ avoidance behaviors in urban parks (Khozaei et al., 2021). Previous studies have revealed that PA impacts mental health (Bélanger et al., 2019). Parental influence was shown to substantially affect children’s perception and participation in PA (Cheung and Chow, 2010). Besides, parental encouragement (Welk et al., 2003) or involvement (Ornelas et al., 2007), as well as role model factors (Bois et al., 2005) in PA, has proved to predict PA among children. Carver et al. (2012) provided statistical evidence that parents’ PA avoidance can affect children’s PA level. Moderate-to-vigorous physical activity (MVPA) is correlated with reducing mental disorders such as depression, anxiety, and mental health improvement (Carson et al., 2016). It has been well-established that outdoor physical activities positively affect peoples’ mental health more than indoor PA (Thompson Coon et al., 2011; Bailey et al., 2018).

Spending time outdoors has been associated with children’s attention improvement (Ulset et al., 2017). Based on these ideas, we raised the following research hypotheses:

H1. Perceived risk (perceived vulnerability and severity) affects parental PA avoidance.

H2. Parents’ stress affects parental PA avoidance.

H3. Parental PA avoidance affects children’s PA.

H4. Children’s PA affects their mental health and wellbeing.

## Materials and Methods

**FIGURE 1:**
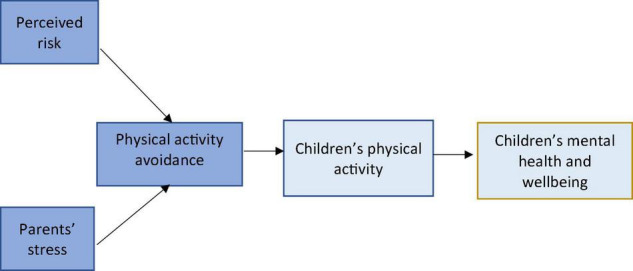
The conceptual research model and the relationship between variables of interest. Dark blue, parents’ related variables; light blue, children’s related variables.

In this study, we aimed to examine the impact of parental stress and perceived risk on children’s avoidance of PA and, consequently, on children’s PA and wellbeing levels. Understanding the contributors to children’s wellbeing during pandemic disease is the first critical step in contributing to children’s health during epidemic diseases. We defined four hypotheses (H1–H4) addressing the conceptual research model (Figure 1).

### Participants

While, currently, several countries have celebrated the end of the COVID-19 pandemic in their countries, Iran is still challenging with a large number of reported death and lockdowns. In October 2021, the school children started their education online for the third year. As Iranian school children are among those who have experienced the most extended lockdowns, examining their PA level and mental health can provide valuable information in developing current literature. Data were collected from primary schools in Kerman city, located southern part of Iran. We selected Kerman city as the capital of one of the largest provinces in Iran, which, alongside the other cities, has experienced the long-term lockdowns and the closing of schools. In total, 306 questionnaires were filled, of which 276 returned online were usable. The responses that either children or their parents left their part blank were removed from the data analysis (30 in total).

Children aged 10–12 years were recruited from eight primary schools in Kerman, Iran. Children of this age generally have permission for independent mobility in short distances from home on parents’ consent. The results of the independent-samples Student’s *t*-test, *t*(274) = 1.25, *p* = 0.211, indicated that there was not any significant difference between the age of male and female students. As parents’ data, we used either the data of the father or the mother of the children, i.e., the parents decided this on their own. In the end, this yields an unbalanced gender ratio with clearly fewer fathers taking part (fathers: *n* = 56; mothers: *n* = 220). The parents aged between 30 and 52 years with a mean age of 42.3 years. The parents had at least one child aged between 10 and 12 years. Those with more than a child of this age were allowed to fill out another questionnaire, respectively. Table 1 presents the frequencies and percentages of school students’ age by their parents’ age group levels. The parents lived with their child/children and were considered their guardians. None of the children suffered from any reported disabilities or critical health problems.

**TABLE 1:** Frequencies and percentages of school students’ age crossed by levels of their parents’ age group levels.

			School students’ age			Total
			10 years	11 years	12 years	
Parents’	30–40	Count	43	30	20	93
age group		%	46.2%	32.3%	21.5%	100.0%
	41–50	Count	66	36	20	122
		%	54.1%	29.5%	16.4%	100.0%
	51 and above	Count	29	18	14	61
		%	47.5%	29.5%	23.0%	100.0%
Total		Count	138	84	54	276
		%	50.0%	30.4%	19.6%	100.0%

### Instruments

As was mentioned earlier, this study aimed to examine factors contributing to children’s wellbeing during the COVID-19 pandemic. Parents answered questions on the perceived risk of COVID-19, stress, PA avoidance, and children responded with questions measuring their PA level and wellbeing. This study explains the measurement scales for each variable.

#### Perceived Risk

Perceived risk consists of two constructs (i.e., perceived severity and perceived efficacy) (Bults et al., 2011). The perceived risk of pandemic disease and, accordingly, peoples’ behavioral response has been examined by some studies during H1N1 influenza waves (e.g., the 2009 swine flu pandemic, the 1977 “Russian” flu pandemic, or the 1918 “Spanish” flu pandemic) or the 2003 severe acute respiratory syndrome (SARS) distribution. To measure the perceived risk, the study relied on the questionnaire by Bults et al. (2011), which was applied to measure knowledge and perceived risk of H1N1 in the Netherlands. Theoretically, the questionnaire relies on the Protection Motivation Theory (PMT) (Norman P. et al., 2015) and the Health Belief Model (HBM) (Champion and Skinner, 2008) for the development of the constructs. The parents were asked to answer three questions on (1) how do they consider the severity of COVID-19 in general, (2) the children getting sick, and (3) its harmfulness for their children. The parents rated the first two questions on a Likert scale from 1 (*not severe*) at all to 5 (*very severe*) and the third question from 1 (*totally disagree*) to 5 (*agree*). Three questions addressed the perceived vulnerability of the COVID-19 pandemic. The parents declared their opinion about the perceived vulnerability of COVID-19 for their children by answering questions addressing the perceived susceptibility of their children against COVID-19, the chance of children getting infected, and the perceived likelihood of them getting infected compared to others on a Likert scale from 1 (*not at all*) to 5 (*very much*). For measuring the perceived risk, the scores of perceived severity and perceived vulnerability were combined.

#### Avoidance of Physical Activity

Parents might apply constrained behaviors to children’s PA when perceiving a possible risk. Constrained behaviors refer to avoidance or defensive actions. This study examines the potential effect of parental stress and perceived risk on children’s PA avoidance, in which parents remove the chance of children’s outdoor PA. Carver et al.’s (2010) items were adapted in this study for measuring PA avoidance. Seven questions examined various types of outdoor PA avoidance: playing alone or with friends in the neighborhood, spending time outdoors, walking or cycling outdoor with alone or friends, and playing alone or with friends in the neighborhood park. They ranked their answer on a Likert scale from 1 (*totally disagree*) to 5 (*totally agree*).

#### Parental Stress

We employed the Depression, Anxiety, and Stress Scale (DASS-21) by Park et al. (2020). The parents were asked to read seven questions and declare how much each statement applied to them during the past week. The questions were as follows: (1) I felt I was close to panic, (2) I was unable to become enthusiastic about anything, (3) I felt I was not worth much as a person, (4) I felt that I was rather touchy, (5) I was aware of the action of my heart in the absence of physical exertion, (6) I felt scared without any good reason, and (7) I felt that life was meaningless. The parents rated their response on a Likert scale from 0 (*did not apply to me at all*) to 3 (*applied to me very much, or most of the tim*e).

#### Physical Activity

For measuring children’s PA level, the International Physical Activity Questionnaire (IPAQ) was used. The short form of the questionnaire contains seven questions addressing the PA level of respondents. The measurement scale has been extensively used worldwide and translated into various languages (refer to “Reliability and validity for 12 countries,” Craig et al., 2003). The questionnaire measures children’s amount and intensity of physical activities (vigorous, moderate, and walking) in the last 7 days. They were required to declare which of the vigorous, moderate, walking, or even sitting they have had in the previous week and how many times. They were also required to specify each activity’s time in one of those days accompanied by information with the respective duration in hours and minutes. The IPAQ has been administered in a series of studies about children’s PA level in COVID-19 (e.g., Zhu et al., 2019; Zhang et al., 2020; Daga et al., 2021), which opens the possibility to compare different studies’ results across different cultures.

#### Children’s Wellbeing and Mental Health

For measuring children’s wellbeing, the KIDSCREEN-10 Index was used in this study (Ravens-Sieberer et al., 2010). The questionnaire has been used widely for measuring children’s wellbeing and mental health. Children were asked to answer ten questions and declare their answers on a Likert scale from 1 (*not at all*) to 5 (*very much*). These questions comprise questions about feeling fit and well, full of energy, sad, lonely, having enough time for themselves, being able to do things they wanted to do, had enough time for something they wanted to do, having fun with friends, being able to pay attention, and being treated fairly by their parents. One question addressing doing well at school was emitted due to COVID-19 lockdowns and schools closing at the time of data collection.

### Procedure

This study has concentrated on second-part primary school students between 10 and 12 years. Based on the official statistics in 2021, the total population of primary school students aged between 10 and 12 years has been around 2,152. The statistics derived from the office of education and training in Kerman revealed that there are currently 138 primary schools (70 girls’ schools and 68 boys’ schools), either publicly or privately run. These schools have been distributed within five municipality regions of the city. A probabilistic sample of 276 students was drawn from 25 schools that accepted collaborating in data collection. Upon the approval of the school, the online questionnaire link was distributed in social media groups of parents of students. Each questionnaire consisted of two sections that had to be filled by parents and their children separately. Both parents and children were provided with an online consent form. They have assured the confidentiality of their response and the academic aim of the research. In the online consent form provided at the beginning of the questionnaire, the parents were asked to confirm their interest in attending the survey as well as giving permission for the participation of their children in the survey. Apart from providing their demographic information, parents were asked to answer questions on the perceived severity of COVID-19, their stress level, and physical activities avoidance.

Similarly, children responded to their age and gender questions, followed by questions on their PA level and perceived health and wellbeing. Children were provided with the definition of vigorous and moderate physical activities with various examples. The questionnaire was piloted in a school with 30 participants in October 2020. After the pilot test, the main data collection started on November 1, 2020, and was completed on December 5, 2020, when the last questionnaire was collected. The survey was online for 35 days. The online questionnaire took around 8–13 min for both parents and children to complete. A token of appreciation was posted to the children who participated in the research with a local post service.

### Statistical Analyses (Model Fit)

The proposed model and research hypotheses were tested with the help of SmartPLS 3 (version 3.3.3) software (Ringle et al., 2015). The rationale behind choosing partial least squares (PLS) lies in the exploratory nature of the study. In the proposed model, some variable relationships were not tested previously (e.g., the impact of stress on PA restriction). Besides, PLS is a proper analysis method in examining the indirect effects of variables in the model. For examining the significance of the path coefficient between latent variables, non-parametric bootstrapping with 1,000 replications was applied.

## Results

**FIGURE 2:**
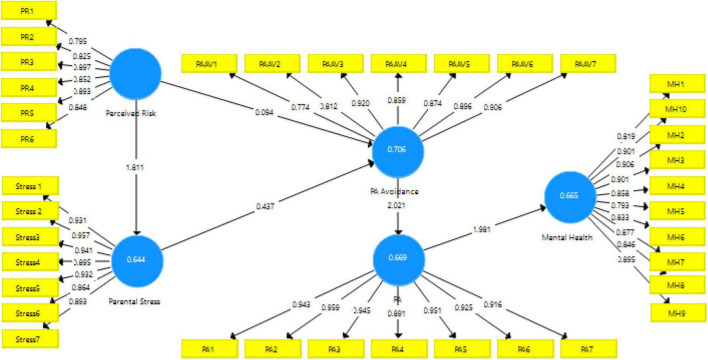
Parent’s influence on children’s PA and mental health during COVID-19.

The purpose of this study was four-fold. First, we explored the effect of perceived risk on avoiding parental PA. Second, we investigated the impact of parents’ stress on the parental avoidance of children’s PA. Third, we studied the effect of parental PA avoidance on children’s PA. Finally, we investigated the effect of children’s PA on their mental health and wellbeing. The model studied in this report, along with its parameter estimations, is displayed in Figure 2.

### Measurement Model Assessment

The proposed model was assessed and employed with a two-stage approach, as Anderson and Gerbing (1988) suggested. Accordingly, we evaluated and presented the Measurement Model and Structural Model. We assessed the measurement model for each latent variable undertaken in the study. We conceptualized it as a reflective measurement model, which allows us to assess its reliability and the convergent and discriminant validity. The reliability of the measurement model was assessed by referring to the Cronbach’s alpha Coefficient, and the Composite Reliability (CR) with the cutoff value of 0.70 was acceptable.

Table 2 indicates that the value of CR and Cronbach’s alpha exceeded the cutoff value of 0.70, suggesting acceptable or better internal consistency reliability. As displayed in Table 2, the Cronbach’s alpha reliability indices for the five components of the model were as follows: mental health (α = 0.962), PA (α = 0.975), PA avoidance (α = 0.943), children’s PA (α = 0.808), mental health (α = 0.932), parental stress (α = 0.968), and perceived risk (α = 0.924). All Cronbach’s alpha reliability indices were higher than the minimum acceptable index of 0.70.

**TABLE 2:** Descriptive statistics and reliability and convergent validity for research constructs.

	Respondent	Source	Number of items	Loadings	Cronbach’s alpha	Rho_A	CR	AVE	Mean and SD
**Physical activity avoidance**	Parents	Carver et al., 2010	7		0.943	0.945	0.954	0.747	(*M* = 18.01, *SD* = 2.97)
I prevent my child from playing alone outdoors in our neighborhood				0.774					
I prevent my child from playing with friends outdoors in our neighborhood				0.812					
I do not allow my child to spend time outside alone,				0.920					
I do not allow my child to walk/ride a bike on the street alone				0.859					
I prevent my child from walking/cycling with friends in our neighborhood				0.874					
I prevent my child from playing alone in our neighborhood park				0.896					
I prevent my child from playing with friends in our neighborhood park				0.906					
**Perceived risk**	Parents	Bults et al., 2011	6		0.924	0.927	0.941	0.727	(*M* = 17.97, SD = 2.99)
*Perceived severity*									
The severity of the Nobel COVID-19 is				0.796					
The severity of getting the COVID-19 for your child/children in the coming year is				0.824					
The COVID-19 is very harmful to your child/children				0.898					
*Perceived vulnerability*				0.852					
How much is your Perceived susceptibility against COVID-19				0.893					
Perceived chance of getting infected next year				0.847					
Perceived chance of getting infected compared to others				0.796					
**Parental stress**	Parents	The (DASS-21)	7		0.968	0.970	0.974	0.840	(*M* = 17.99, *SD* = 3.02)
I felt I was close to panic				0.932					
I was unable to become enthusiastic about anything				0.957					
I felt I was not worth much as a person				0.941					
I felt that I was rather touchy				0.896					
I was aware of the action of my heart in the absence of physical exertion.				0.932					
I felt scared without any good reason				0.862					
I felt that life was meaningless				0.893					
**Children’s physical activity**	Children	International Physical Activity (IPAQ)	7		0.975	0.976	0.979	0.871	(*M* = 17.99, *SD* = 3.00)
Number of days with vigorous physical activities				0.943					
Amount of time spent on vigorous physical activities during 1 day				0.959					
Number of days with moderate physical activities				0.945					
Amount of time spent on moderate physical activities during one day				0.891					
Number of days with at least 10 min walking				0.951					
Amount of time spent on walking during 1 day				0.925					
Amount of time spent sitting on a week day				0.916					
**Children’s wellbeing and mental health**	Children	KIDSCREEN-10	10		0.962	0.968	0.967	0.746	(*M* = 18.02, *SD* = 3.04)
Have you felt fit and well?				0.819					
Have you felt full of energy?				0.901					
Have you felt sad?				0.906					
Have you felt lonely?				0.901					
Have you had enough time for yourself				0.858					
Have you been able to do the things that you want to do in your free time?				0.793					
Have your parent(s) treated you fairly?				0.833					
Have you had fun with your friends?				0.877					
Have you been able to pay attention?				0.846					
In general, how would you say your health is?				0.895					

As evaluated by the average variance extracted (AVE) value for all constructs, the convergent validity exceeded the cutoff value of 0.50. Besides, the outer loading value of all items of each construct was higher than 0.70. Table 2 presents the established reliability and convergent validity for research constructs.

In the next step, we examined the discriminant validity of the constructs. We employed the Fornell-Larcker Criterion and Heterotrait-Monotrait (HTMT) (Voorhees et al., 2016). The accepted HTMT value must be lower than 0.85 or 0.90 (Henseler et al., 2015). Table 3 reveals that discriminant validity was acceptable in the study data. In addition, the criterion developed by Fornell and Larcker (1981) suggests that the square root of AVEs of each construct must be ensured to be greater than the correlation estimate between constructs. Table 4 also reveals supporting this criterion and demonstrating the discriminant validity again.

**TABLE 3:** Fornell-Larcker criterion.

	Mental health	PA	PA avoidance	Parental stress	Perceived risk
Mental health	0.864				
PA	0.815	0.933			
PA avoidance	0.818	0.818	0.864		
Parental stress	0.811	0.959	0.824	0.917	
Perceived risk	0.791	0.814	0.760	0.802	0.852

**TABLE 4:** Heterotrait-Monotrait (HTMT) ratio.

	Mental health	PA	PA avoidance	Parental stress	Perceived risk
Mental health					
PA	0.827				
PA avoidance	0.847	0.851			
Parental stress	0.826	0.987	0.860		
Perceived risk	0.823	0.855	0.812	0.846	

### Structural Model Assessment

To examine the structural model, two preliminary criteria should be checked: the significance of path coefficients and the value of *R*^2^ coefficients for endogenous constructs. Chin (1998) suggested that the values of 0.67, 0.33, and 0.19 suggest substantial, moderate, and weak measures of *R*^2^, respectively. In this study, *R*^2^ for perceived parental stress, PA avoidance, PA, and mental health were 0.644, 0.706, 0.669, and 0.655, respectively, which are relatively high and acceptable values.

Table 5 presents the evaluation of the research hypothesis and path coefficients. There are four hypotheses in this study addressing the measurement model.

**TABLE 5:** Standardized regression weights (along with the 95% confidence interval).

	Paths	*M*	*SD*	*t*-value	*p*	Standardized regression weight	
						2.5%	97.5%
PA→Mental health	0.815	0.819	0.017	47.958	<0.001	0.783	0.848
PA avoidance→PA	−0.818	0.820	0.020	41.301	<0.001	0.778	0.855
Parental stress→PA avoidance	0.600	0.591	0.053	11.322	0.004	0.483	0.689
Perceived risk→PA avoidance	0.279	0.291	0.059	4.704	<0.001	0.183	0.402

*Parents’ influence on children’s PA and wellbeing during COVID-19.*

A: The first hypothesis examined the impact of perceived risk on PA avoidance. The result of the study revealed that parents’ perceived risk significantly affected parental PA avoidance (β = 0.279, *t* = 4.704, *p* < 0.001). Thus, the first directional hypothesis “Perceived risk affected parental avoidance of PA” was supported. Hence, it was statistically shown that the perceived severity and vulnerability of pandemic diseases such as COVID-19 could significantly affect parents’ constrained behaviors.

B: The second hypothesis examined if parents’ stress significantly affected parental PA avoidance (β = 0.600, *t* = 11.322, *p* < 0.004). Thus, the second directional hypothesis “Parents’ stress affected parental PA avoidance” was supported. It suggests that during the COVID-19 pandemic, the higher stress parents experience, the more they might prevent their children from spending time outdoors.

C: The third hypothesis tested the effect of parental PA avoidance on the total level of PA among children aged 10–12 years during the COVID-19 pandemic. Parental PA avoidance had a negative and significant effect on children’s PA (β = −0.818, *t* = 41.301, *p* < 0.001). Thus, the third directional hypothesis “Parental PA avoidance affected children’s PA” was supported.

D: The fourth hypothesis examined the impact of children’s PA on their perceived wellbeing. Children’s PA significantly affected their mental health and wellbeing (β = 0.815, *t* = 47.958, *p* = 0.001). Thus, the fourth directional hypothesis “Children’s PA affected their mental health and wellbeing” was supported. This finding is significant as it suggests that the long-term lockdowns and the lack of PA have affected them negatively, although families stayed at home given the parents’ perception that the home is the safest place to keep children healthy. Children who had the chance of higher PA perceived themselves as healthier.

### Model Fit Indices

The Standardized Root Mean Square Residual (SRMR), which “measures the difference between the observed correlation matrix and the model-implied correlation matrix” (Garson, 2016, p. 68), should be ≤0.8 to infer the model’s fit. Our study’s SRMR of 0.075 indicated that this model enjoyed a good fit. The Squared Euclidean Distance (d_ULS) and Geodesic Distance (d_G) are exact fit indices that, similar to SRMR, measure any significant difference between the observed and model-implied correlation matrices. However, unlike SRMR, which measures the residuals, d_ULS and d_G compute the distances between the two matrices (Hair et al., 2017). Overall, this model enjoyed a good fit.

### Differences in Parental Stress and Children’s Mental Health

The housing type in this research was confined to the apartment and single-story houses, which generally have a private yard used by a single family, i.e., all located in Kerman/Iran. Except for Afghan migrants, these types of accommodation are typically occupied by a single-family. However, in some other cases, extra members of families might coexist in the same house. An independent samples *t*-test analysis was conducted, yielding significant differences between the mental health of children living in an apartment and the single-story homes, *t*(276) = 19.84, *p* < 0.02, Cohen’s *d* = 0.435. The children living in single-story houses (*M* = 31, *SD* = 10.92) considered themselves healthier compared to those living in the apartments (*M* = 12.11, *SD* = 4.81), all measured during COVID-19 pandemic lockdowns. Interestingly, parental stress of those living in apartments (*M* = 15.08, *SD* = 8.28) was also significantly [*t*(276) = 13.46, *p* < 0.01, Cohen’s *d* = 0.643] higher than those who lived in single-story homes (*M* = 4.25, *SD* = 4.83).

Besides, we revealed a significant difference in the stated mental health of children whose parents had job security compared with those who did not have *t*(276) = 13.66, *p* < 0.07, *n.s.*, Cohen’s *d* = 0.821. Children whose parents had a secure job during COVID-19 had a far better mental health (*M* = 29.67, *SD* = 11.89) comparing their counterparts (*M* = 13.51, *SD* = 7.26). Job security was also significantly associated with parents’ perceived stress, *t*(276) = 21.88, *p* < 0.00, Cohen’s *d* = 0.621. Those with insecure jobs experienced higher stress (*M* = 17.53, *SD* = 6.05) comparing with parents with secure jobs (*M* = 3.59, *SD* = 4.10). Through running a series of ANOVA tests, we found that children’s mental health might differ when they do not have or have other siblings, *F*(4,272) = 20.52, *p* = 0.03, Cohen’s *d* = 0.321. Those with one brother or sister reported the best mental health status (*M* = 22.54, *SD* = 1.00), and children with four or more siblings were reported with the poorest mental health status (*M* = 10.00, *SD* = 2.04) during the COVID-19 lockdowns.

The children’s mental health status was also significantly different between those residing in more or less crowded living environments, *F*(4,272) = 41.48, *p* = 0.02, Cohen’s *d* = 0.468. Children who were living in a family of four members were associated with the best mental health (*M* = 34.13, *SD* = 12.53). Living in populated families was also associated with a significant rise in parents’ stress, *F*(4,272) = 24.42, *p* < 0.0001, Cohen’s *d* = 0.786. The highest reported stress was associated with those living in families with 6+ members (*M* = 17.06, *SD* = 7.12). History of death or hospitalization of relatives or family members due to COVID-19 was significantly associated with parents’ stress *t*(276) = 15.60, *p* < 0.0001, Cohen’s *d* = 0.587. Children with no history of such personal experiences with COVID-19-related health issues in their families and relatives showed a much more balanced mental health status (*M* = 27.80, *SD* = 11.93) than their counterparts (*M* = 14.67, *SD* = 9.05).

## Discussion and Conclusion

It was late 2019 that people worldwide heard about an unknown, deadly virus that presumably originated from China for the first time. It did not take long that the transmitted virus outside the China borders brought about similar symptoms, death, and fear globally. Accordingly, staying home and keeping social distancing turned into a worldwide slogan, and attitude and respective measures were implemented all over the world. Coping with the lengthy lockdowns and curfews, fear of death, losing the job, and uncertainty about the future greatly stressed people on a global scale.

COVID-19 posed numerous challenges for people confronted with restrictions, lockdowns, and quarantine situations (Grossman et al., 2020; Picaza Gorrochategi et al., 2020; Zhu et al., 2020).

Parenting is taken as concerns about financial support of family, training, and taking care of children’s education, concerns about job stability, and tolerating working conditions. Parenting has always been considered a difficult task, yet the burden of COVID-19 faced parents with a more significant challenge (Cluver et al., 2020; Griffith, 2020). Job insecurity concerns during COVID-19, providing children with proper mobile electrical devices for online learning, responding to children’s new demands, working from home have been all-new aspects of parenting for children. There is no wonder that several studies have canonized the parents’ stress during COVID-19 (Brown et al., 2020; Calvano et al., 2021; Chung et al., 2020; Xu et al., 2020). The impact of parents’ emphasis on various aspects of children’s life is becoming increasingly difficult to ignore.

In this study, we aimed to investigate the relationship between five latent constructs: perceived risk of COVID-19, avoidance of PA, parental stress, children’s PA, and children’s mental health. Four hypotheses were developed and tested using SmartPLS 3. Through a consequential model, we examined some factors contributing to children’s mental health. Results of the study provided empirical evidence for the hypothesized relationship between the supposed latent constructs.

The findings supported the first hypothesis. Similar to Carver et al. (2012), it appears that perceived risk affects parental PA avoidance. Based on the literature, parents prefer to avoid their adolescent or children’s PA outdoor in any risky conditions such as neighborhood safety (Weir et al., 2006), traffic (Timperio et al., 2004; Carver et al., 2010), and danger of strangers (Carver et al., 2010). Notably, for the PA level, we referred to a well-established instrument (IPAQ) for which we could not rely on Persian norms as this instrument is not yet validated for Iran. However, *a posteriori* analyses for our sample revealed a reliable scale also for our adapted version for the Persian language.

Parents’ stress was revealed to affect children’s PA avoidance significantly. Accordingly, the second hypothesis was also supported. There might be several explanations for this result. First, previous studies have already shown the negative impact of stress on ones’ PA. For example, Salmon (2001) asserted that “people who are less disturbed by stress might simply be more ready to take up exercise training” (p. 46). Hence, the inactivity of stressed parents might influence PA restrictions for their children. Second, parents with higher stress levels might have more negative thoughts and will likely expect bad events. This might explain the higher probabilities of restrictive behaviors on children’s spending time outdoor.

Our findings supported the third hypothesis as well. Parents’ PA avoidance proved to have a strong negative effect on children’s overall PA level. We could demonstrate that children with parents who applied more restriction rules on children’s PA also showed lower PA levels. Previous studies argue that spending time outdoor can positively affect youth’s level of PA (Schaefer et al., 2014; Gray et al., 2015). Accordingly, they also justify that avoidance of children from the time of expenditures’ outdoor leads to the low level of children’s PA. This result is consistent with the findings of other studies (Timperio et al., 2004; Weir et al., 2006).

The fourth hypothesis examined the impact of children’s PA on their mental health and wellbeing. The result of the study supported the research hypothesis. Children with less PA level considered themselves less healthy, and their overall wellbeing was less than those with higher PA levels. It is widely accepted that PA is associated with children’s health (Christiansen et al., 2018; Bélanger et al., 2019; García-Hermoso et al., 2020b) and children’s activity limitation increases the chance of being overweight (Holderness et al., 2017). The current findings support those previous findings.

Taken as a whole, the result of this study highlights the importance of PA on children’s mental health. It also canonizes the role of parents on children’s PA level during pandemic disease. Before COVID-19, children were performing PA in various, freely choosing contexts. Structured activities and sports courses in the school or walking and cycling to get to the school are all essential measures to add active hours to children’s lifestyle. Physical playing indoors or outdoors and engaging in group games are typically also part of children’s PA. COVID-19 made many children’s routine PA impossible or even explicitly prohibited them. Parents, as the most critical gatekeepers, significantly controlled children’s PA, especially in pandemic times. Nowadays, the children’s average physical activities might not even meet the recommended PA daily level (Table 6). This study intriguingly demonstrates the adverse effects of children’s less PA during the long-term stressful periods such as epidemic diseases. It shed light on an essential fact that the children who had less PA in the last 7 days also perceived themselves as less healthy. As the current pandemic situation has limited the children’s average time to spend time outdoors, it is essential to facilitate access to good physical playing possibilities, e.g., providing gaming devices to encourage physical play on safe outdoor fields. All these issues should be taken very seriously by parents, by caring institutions, and, of course, by state officials.

**TABLE 6:** Recommended physical activity (PA) based on age group.

Age group	Physical activity duration
Preschool-aged children (3–5 years)	Physical activity every day throughout the day. Active play through a variety of enjoyable physical activities.
Children and adolescents (6–17 years)	60 min (1 h) or more of moderate-to-vigorous intensity physical activity daily. A variety of enjoyable physical activities.
Adults (18–64 years)	At least 150 min a week of moderate intensity activity such as brisk walking. At least 2 days a week of activities that strengthen muscles.

In canonizing the parental stress and children’s mental health, we further compared the respondents regarding the demographic background and living environment. We found that housing type, parents’ job security, number of siblings, number of members living together in-home, and history of death or hospitalization of relatives or family members due to COVID-19 were associated with parents’ stress and children’s mental health. Further studies might consider other factors such as neighborhood condition, housing area and the number of rooms, and the possible connection with nature as factors that can affect the parents’ stress and children’s mental health. Besides, the comparative study of children’s mental health in rural areas and urban environments might provide valuable information.

Our study is among the very first research approaches examining the impact of parents’ stress on PA restrictions. We believe that understanding factors contributing to children’s mental health and wellbeing is essential in times of continuing lockdowns. Of course, future research has to test also the developments and adaptations taking place during such a long-term crisis similar to COVID-19 because our present study only provides a single-shot measurement of the target variables. Further studies might also canonize the role of parenting strategies and the management of children’s PA during such critical periods of life on children’s overall wellbeing. Insights generated from such studies will be an important learning lesson for other crises to come in the future.

## Research Limitations

Due to the closing of schools, we did not have access to an extremely large number of participants. We were confined to schools that had social media groups for students and parents. Some of the school principals also disagreed in distributing the required online link among the students and respective explanations of research aims for their parents. These are challenges that increase the difficulties in conducting such studies in this area under the circumstances of a pandemic.

## Data Availability Statement

The raw data supporting the conclusions of this article will be made available by the authors, without undue reservation.

## Ethics Statement

Ethical review and approval was not required for the study on human participants in accordance with the local legislation and institutional requirements. Written informed consent to participate in this study was provided by the participants’ legal guardian/next of kin.

## Author Contributions

FK analyzed the data. Both authors were involved in the research design, manuscript preparation, and approved the submitted version.

## Conflict of Interest

The authors declare that the research was conducted in the absence of any commercial or financial relationships that could be construed as a potential conflict of interest.

## Publisher’s Note

All claims expressed in this article are solely those of the authors and do not necessarily represent those of their affiliated organizations, or those of the publisher, the editors and the reviewers. Any product that may be evaluated in this article, or claim that may be made by its manufacturer, is not guaranteed or endorsed by the publisher.

## References

[B1] Anderson J. C. Gerbing D. W. (1988). Structural equation modeling in practice: a review and recommended two-step approach. *Psychol. Bull.* 103 411–423. 10.1037/0033-2909.103.3.411

[B2] Bailey A. W. Allen G. Herndon J. Demastus C. (2018). Cognitive benefits of walking in natural versus built environments. *World Leis. J.* 60 293–305. 10.1080/16078055.2018.1445025

[B3] Bedell G. Coster W. Law M. Liljenquist K. Kao Y.-C. Teplicky R. (2013). Community participation, supports, and barriers of school-age children with and without disabilities. *Arch. Phys. Med. Rehabil.* 94 315–323. 10.1016/j.apmr.2012.09.024 23044364

[B4] Bélanger M. Gallant F. Doré I. O’Loughlin J. L. Sylvestre M.-P. Abi Nader P. (2019). Physical activity mediates the relationship between outdoor time and mental health. *Prev. Med. Rep.* 16:101006. 10.1016/j.pmedr.2019.101006 31720202 PMC6838503

[B5] Bois J. E. Sarrazin P. G. Brustad R. J. Trouilloud D. O. Cury F. (2005). Elementary schoolchildren’s perceived competence and physical activity involvement: the influence of parents’ role modelling behaviours and perceptions of their child’s competence. *Psychol. Sport Exerc.* 6 381–397. 10.1016/j.psychsport.2004.03.003

[B6] Brown S. M. Doom J. R. Lechuga-Pena S. Watamura S. E. Koppels T. (2020). Stress and parenting during the global COVID-19 pandemic. *Child Abuse Negl.* 110(Pt. 2):104699. 10.1016/j.chiabu.2020.104699 32859394 PMC7440155

[B7] Bults M. Beaujean D. J. de Zwart O. Kok G. van Empelen P. van Steenbergen J. E. (2011). Perceived risk, anxiety, and behavioural responses of the general public during the early phase of the Influenza A (H1N1) pandemic in the Netherlands: results of three consecutive online surveys. *BMC Public Health* 11:2. 10.1186/1471-2458-11-2 21199571 PMC3091536

[B8] Calvano C. Engelke L. Di Bella J. Kindermann J. Renneberg B. Winter S. M. (2021). Families in the COVID-19 pandemic: parental stress, parent mental health and the occurrence of adverse childhood experiences”results of a representative survey in Germany. *Eur. Child Adolesc. Psychiatry* 1–13. 33646416 10.1007/s00787-021-01739-0 PMC7917379

[B9] Carson V. Hunter S. Kuzik N. Gray C. E. Poitras V. J. Chaput J.-P. (2016). Systematic review of sedentary behaviour and health indicators in school-aged children and youth: an update. *Appl. Physiol. Nutr. Metab.* 41 240–265. 10.1139/apnm-2015-0630 27306432

[B10] Carver A. Timperio A. Hesketh K. Crawford D. (2010). Are children and adolescents less active if parents restrict their physical activity and active transport due to perceived risk? *Soc. Sci. Med.* 70 1799–1805. 10.1016/j.socscimed.2010.02.010 20347200

[B11] Carver A. Timperio A. Hesketh K. Crawford D. (2012). How does perceived risk mediate associations between perceived safety and parental restriction of adolescents’ physical activity in their neighborhood? *Int. J. Behav. Nutr. Phys. Act.* 9:57. 10.1186/1479-5868-9-57 22607169 PMC3458944

[B12] Cavill N. Biddle S. Sallis J. F. (2001). Health enhancing physical activity for young people: statement of the United Kingdom expert consensus conference. *Pediatr. Exerc. Sci.* 13 12–25.

[B13] Champion V. L. Skinner C. S. (2008). The health belief model. *Health Behav. Health Educ.* 4 45–65.

[B14] Cheung P. Y. P. Chow B. C. (2010). Parental mediatory role in children’s physical activity participation. *Health Educ.* 110 351–366. 10.1108/09654281011068513

[B15] Chin W. W. (1998). The partial least squares approach to structural equation modeling. *Mod. Methods Bus. Res.* 295 295–336.

[B16] Chong K. H. Parrish A.-M. Cliff D. P. Kemp B. J. Zhang Z. Okely A. D. (2020). Changes in physical activity, sedentary behavior and sleep across the transition from primary to secondary school: a systematic review. *J. Sci. Med. Sport* 23 498–505. 10.1016/j.jsams.2019.12.002 31848107

[B17] Christiansen L. B. Lund-Cramer P. Brondeel R. Smedegaard S. R. Holt A.-D. Skovgaard T. (2018). Improving children’s physical self-perception through a school-based physical activity intervention: the Move for Well-being in School study. *Ment. Health Phys. Act.* 14 31–38. 10.1016/j.mhpa.2017.12.005

[B18] Chung G. Lanier P. Wong P. Y. J. (2020). Mediating effects of parental stress on harsh parenting and parent-child relationship during coronavirus (COVID-19) pandemic in Singapore. *J. Fam. Violence* 1–12. 10.1007/s10896-020-00200-1 32895601 PMC7467635

[B19] Cluver L. Lachman J. M. Sherr L. Wessels I. Krug E. Rakotomalala S. (2020). Parenting in a time of COVID-19. *Lancet* 395:e64. 10.1016/S0140-6736(20)30736-4 32220657 PMC7146667

[B20] Craig C. L. Marshall A. L. Sjöström M. Bauman A. E. Booth M. L. Ainsworth B. E. (2003). International physical activity questionnaire: 12-country reliability and validity. *Med. Sci. Sports Exerc.* 35 1381–1395. 10.1249/01.Mss.0000078924.61453.Fb 12900694

[B21] Daga F. A. Agostino S. Peretti S. Beratto L. (2021). COVID-19 nationwide lockdown and physical activity profiles among North-western Italian population using the International Physical Activity Questionnaire (IPAQ). *Sport Sci. Health* 17 459–464. 10.1007/s11332-021-00745-8 33688376 PMC7931493

[B22] Drouin M. McDaniel B. T. Pater J. Toscos T. (2020). How parents and their children used social media and technology at the beginning of the COVID-19 pandemic and associations with anxiety. *Cyberpsychol. Behav. Soc. Netw.* 23 727–736. 10.1089/cyber.2020.0284 32726144

[B23] Fornell C. Larcker D. F. (1981). *Structural Equation Models with Unobservable Variables and Measurement Error: Algebra and Statistics.* Los Angeles, CA: Sage Publications.

[B24] García-Hermoso A. Ezzatvar Y. Ramírez-Vélez R. Olloquequi J. Izquierdo M. (2020a). Is device-measured vigorous-intensity physical activity associated with health-related outcomes in children and adolescents? A systematic review and meta-analysis. *J. Sport Health Sci.* 10 296–307. 10.1016/j.jshs.2020.12.001 33285309 PMC8167335

[B25] García-Hermoso A. Hormazábal-Aguayo I. Fernández-Vergara O. Olivares P. R. Oriol-Granado X. (2020b). Physical activity, screen time and subjective well-being among children. *Int. J. Clin. Health Psychol.* 20 126–134. 10.1016/j.ijchp.2020.03.001 32550852 PMC7296239

[B26] Garson G. D. (2016). *Partial Least Squares: Regression and Structural Equation Models.* Asheboro, NC: Statistical Associates Publishers.

[B27] Gray C. Gibbons R. Larouche R. Sandseter E. B. H. Bienenstock A. Brussoni M. (2015). What is the relationship between outdoor time and physical activity, sedentary behaviour, and physical fitness in children? a systematic review. *J. Environ. Res. Public Health* 12 6455–6474. 10.3390/ijerph120606455 26062039 PMC4483711

[B28] Griffith A. K. (2020). Parental burnout and child maltreatment during the COVID-19 pandemic. *J. Fam. Violence* 1–7. 10.1007/s10896-020-00172-2 32836736 PMC7311181

[B29] Grossman E. S. Hoffman Y. S. G. Palgi Y. Shrira A. (2020). COVID-19 related loneliness and sleep problems in older adults: worries and resilience as potential moderators. *Pers. Individ. Dif.* 168:110371. 10.1016/j.paid.2020.110371 32904342 PMC7455172

[B30] Hair J. Jr. Hult G. T. M. Ringle C. M. Sarstedt M. (2017). *A Primer on Partial Least Squares Structural Equation Modeling (PLS-SEM)*, 1st Edn. Thousand Oaks, CA: SAGE publication.

[B31] Henseler J. Ringle C. M. Sarstedt M. (2015). A new criterion for assessing discriminant validity in variance-based structural equation modeling. *J. Acad. Mark. Sci.* 43 115–135. 10.1007/s11747-014-0403-8

[B32] Hill D. C. Moss R. H. Sykes-Muskett B. Conner M. O’Connor D. B. (2018). Stress and eating behaviors in children and adolescents: systematic review and meta-analysis. *Appetite* 123 14–22. 10.1016/j.appet.2017.11.109 29203444

[B33] Hillman M. (2006). Children’s rights and adults’ wrongs. *Child Geogr.* 4 61–67. 10.1080/14733280600577418

[B34] Holderness H. Chin N. Ossip D. J. Fagnano M. Reznik M. Halterman J. S. (2017). Physical activity, restrictions in activity, and body mass index among urban children with persistent asthma. *Ann. Allergy Asthma Immunol.* 118 433–438. 10.1016/j.anai.2017.01.014 28268134 PMC5385295

[B35] Karsten L. (2005). It all used to be better? Different generations on continuity and change in urban children’s daily use of space. *Child Geogr.* 3 275–290. 10.1080/14733280500352912

[B36] Kato N. Yanagawa T. Fujiwara T. Morawska A. (2015). Prevalence of children’s mental health problems and the effectiveness of population-level family interventions. *J. Epidemiol.* 25 507–516. 10.2188/jea.JE20140198 26250791 PMC4517988

[B37] Khozaei F. Kim M. J. Nematipour N. Ali A. (2021). The impact of perceived risk and disease prevention efficiency on outdoor activities and avoidance behaviors in the urban parks during COVID 19 pandemic. *J. Facil. Manag.* 19 553–568. 10.1108/JFM-09-2020-0065

[B38] Leary S. D. Ness A. R. Smith G. D. Mattocks C. Deere K. Blair S. N. (2008). Physical activity and blood pressure in childhood. *Hypertension* 51 92–98. 10.1161/HYPERTENSIONAHA.107.099051 18071055

[B39] Limbers C. A. McCollum C. Greenwood E. (2020). Physical activity moderates the association between parenting stress and quality of life in working mothers during the COVID-19 pandemic. *Ment. Health Phys. Act.* 19:100358. 10.1016/j.mhpa.2020.100358 33072187 PMC7548083

[B40] Loucaides C. A. Jago R. (2008). Differences in physical activity by gender, weight status and travel mode to school in Cypriot children. *Prev. Med.* 47 107–111. 10.1016/j.ypmed.2008.01.025 18367241

[B41] Norman Ã Berlin A. Sundblom E. Elinder L. S. Nyberg G. (2015). Stuck in a vicious circle of stress. Parental concerns and barriers to changing children’s dietary and physical activity habits. *Appetite* 87 137–142. 10.1016/j.appet.2014.12.20 25542774

[B42] Norman P. Boer H. Seydel E. R. Mullan B. (2015). “Protection motivation theory,” in *Predicting and Changing Health Behavior*, eds Conner M. Norman P. (Buckingham: Open University Press), 70–106.

[B43] Ornelas I. J. Perreira K. M. Ayala G. X. (2007). Parental influences on adolescent physical activity: a longitudinal study. *Int. J. Behav. Nutr. Phys. Act.* 4:3. 10.1186/1479-5868-4-3 17274822 PMC1805507

[B44] Park S. H. Song Y. J. C. Demetriou E. A. Pepper K. L. Thomas E. E. Hickie I. B. (2020). Validation of the 21-item depression, anxiety, and stress scales (DASS-21) in individuals with autism spectrum disorder. *Psychiatry Res.* 291:113300. 10.1016/j.psychres.2020.113300 32763554

[B45] Parola A. Rossi A. Tessitore F. Troisi G. Mannarini S. (2020). Mental health through the COVID-19 quarantine: a growth curve analysis on Italian young adults. *Front. Psychol.* 11:567484. 10.3389/fpsyg.2020.567484 33132973 PMC7566042

[B46] Patel V. Flisher A. J. Hetrick S. McGorry P. (2007). Mental health of young people: a global public-health challenge. *Lancet* 369 1302–1313. 10.1016/S0140-6736(07)60368-7 17434406

[B47] Patrick S. W. Henkhaus L. E. Zickafoose J. S. Lovell K. Halvorson A. Loch S. (2020). Well-being of parents and children during the COVID-19 pandemic: a national survey. *Pediatrics* 146:e2020016824. 10.1542/peds.2020-016824 32709738

[B48] Picaza Gorrochategi M. Eiguren Munitis A. Dosil Santamaria M. Ozamiz Etxebarria N. (2020). Stress, anxiety, and depression in people aged over 60 in the COVID-19 outbreak in a sample collected in Northern Spain. *Am. J. Geriatr. Psychiatry* 28 993–998. 10.1016/j.jagp.2020.05.022 32576424 PMC7261426

[B49] Ravens-Sieberer U. Erhart M. Rajmil L. Herdman M. Auquier P. Bruil J. (2010). Reliability, construct and criterion validity of the KIDSCREEN-10 score: a short measure for children and adolescents’ well-being and health-related quality of life. *Qual. Life Res.* 19 1487–1500. 10.1007/s11136-010-9706-5 20668950 PMC2977059

[B50] Ridgers N. D. Stratton G. Fairclough S. J. (2006). Physical activity levels of children during school playtime. *Sports Med.* 36 359–371. 10.2165/00007256-200636040-0000 16573359

[B51] Ringle C. M. Wende S. Becker J.-M. (2015). *SmartPLS 3. Bönningstedt: SmartPLS.* Available online at: http://www.smartpls.com (accessed December 10, 2021).

[B52] Sallis J. F. Prochaska J. J. Taylor W. C. (2000). A review of correlates of physical activity of children and adolescents. *Med. Sci. Sports Exerc.* 32 963–975. 10.1097/00005768-200005000-00014 10795788

[B53] Salmon J. Timperio A. Cleland V. Venn A. (2005). Trends in children’s physical activity and weight status in high and low socio-economic status areas of Melbourne, Victoria, 1985-2001. *J. Public Health* 29 337–342. 10.1111/j.1467-842x.2005.tb00204.x 16222931

[B54] Salmon P. (2001). Effects of physical exercise on anxiety, depression, and sensitivity to stress: a unifying theory. *Clin. Psychol. Rev.* 21 33–61. 10.1016/s0272-7358(99)00032-x 11148895

[B55] Schaefer L. Plotnikoff R. C. Majumdar S. R. Mollard R. Woo M. Sadman R. (2014). Outdoor time is associated with physical activity, sedentary time, and cardiorespiratory fitness in youth. *J. Pediatr.* 165 516–521. 10.1016/j.jpeds.2014.05.029 25043155

[B56] Schoeppe S. Duncan M. J. Badland H. Oliver M. Curtis C. (2013). Associations of children’s independent mobility and active travel with physical activity, sedentary behaviour and weight status: a systematic review. *J. Sci. Med. Sport* 16 312–319. 10.1016/j.jsams.2012.11.001 23219100

[B57] Shoesmith A. Hall A. Hope K. Sutherland R. Hodder R. K. Trost S. G. (2020). Associations between in-school-hours physical activity and child health-related quality of life: a cross-sectional study in a sample of Australian primary school children. *Prev. Med. Rep.* 20:101179. 10.1016/j.pmedr.2020.101179 PMC7452118 32884897

[B58] Thompson Coon J. Boddy K. Stein K. Whear R. Barton J. Depledge M. H. (2011). Does participating in physical activity in outdoor natural environments have a greater effect on physical and mental well-being than physical activity indoors? A systematic review. *Environ. Sci. Technol.* 45 1761–1772. 10.1021/es102947t 21291246

[B59] Thomson K. C. Richardson C. G. Gadermann A. M. Emerson S. D. Shoveller J. Guhn M. (2019). Association of childhood social-emotional functioning profiles at school entry with early-onset mental health conditions. *JAMA Netw. Open* 2:e186694. 10.1001/jamanetworkopen.2018.6694 30646194 PMC6324314

[B60] Timperio A. Crawford D. Telford A. Salmon J. (2004). Perceptions about the local neighborhood and walking and cycling among children. *Prev. Med.* 38 39–47. 10.1016/j.ypmed.2003.09.026 14672640

[B61] Towns N. D’Auria J. (2009). Parental perceptions of their child’s overweight: an integrative review of the literature. *J. Pediatr. Nurs.* 24 115–130. 10.1016/j.pedn.2008.02.032 19268233

[B62] Ulset V. Vitaro F. Brendgen M. Bekkhus M. Borge A. I. H. (2017). Time spent outdoors during preschool: links with children’s cognitive and behavioral development. *J. Environ. Psychol.* 52 69–80. 10.1016/j.jenvp.2017.05.007

[B63] Valentine G. McKendrck J. (1997). Children’s outdoor play: exploring parental concerns about children’s safety and the changing nature of childhood. *Geoforum* 28 219–235. 10.1016/S0016-7185(97)00010-9

[B64] Vloet T. D. Konrad K. Herpertz-Dahlmann B. Polier G. G. Günther T. (2010). Impact of anxiety disorders on attentional functions in children with ADHD. *J. Affect. Disord.* 124 283–290. 20064664 10.1016/j.jad.2009.11.017

[B65] Voorhees C. M. Brady M. K. Calantone R. Ramirez E. (2016). Discriminant validity testing in marketing: an analysis, causes for concern, and proposed remedies. *J. Acad. Mark. Sci.* 44 119–134. 10.1007/s11747-015-0455-4

[B66] Weir L. A. Etelson D. Brand D. A. (2006). Parents’ perceptions of neighborhood safety and children’s physical activity. *Prev. Med.* 43 212–217. 10.1016/j.ypmed.2006.03.024 16712912

[B67] Welk G. J. Wood K. Morss G. (2003). Parental influences on physical activity in children: an exploration of potential mechanisms. *Pediatr. Exerc. Sci.* 15 19–33. 10.1093/deafed/ent033 23833318

[B68] Wohlfarth R. Mutschler B. Beetz A. Kreuser F. Korsten-Reck U. (2013). Dogs motivate obese children for physical activity: key elements of a motivational theory of animal-assisted interventions. *Front. Psychol.* 4:796. 10.3389/fpsyg.2013.00796 24194726 PMC3810595

[B69] Wright K. E. Furzer B. J. Licari M. K. Thornton A. L. Dimmock J. A. Naylor L. H. (2019). Physiological characteristics, self-perceptions, and parental support of physical activity in children with, or at risk of, developmental coordination disorder. *Res. Dev. Disabil.* 84 66–74. 10.1016/j.ridd.2018.05.013 29914720

[B70] Wu M. Xu W. Yao Y. Zhang L. Guo L. Fan J. (2020). Mental health status of students’ parents during COVID-19 pandemic and its influence factors. *Gen. Psychiatr.* 33:e100250. 10.1136/gpsych-2020-100250 34192232 PMC7387315

[B71] Xu Y. Wu Q. Levkoff S. E. Jedwab M. (2020). Material hardship and parenting stress among grandparent kinship providers during the COVID-19 pandemic: the mediating role of grandparents’ mental health. *Child Abuse Negl.* 110:104700. 10.1016/j.chiabu.2020.104700 32854948 PMC7444952

[B72] Yeasmin S. Banik R. Hossain S. Hossain M. N. Mahumud R. Salma N. (2020). Impact of COVID-19 pandemic on the mental health of children in Bangladesh: a cross-sectional study. *Child Youth Serv. Rev.* 117:105277. 10.1016/j.childyouth.2020.105277 32834275 PMC7387938

[B73] Zhang X. Zhu W. Kang S. Qiu L. Lu Z. Sun Y. (2020). Association between physical activity and mood states of children and adolescents in social isolation during the COVID-19 Epidemic. *Int. J. Environ. Res. Public Health* 17:7666. 10.3390/ijerph17207666 33096659 PMC7589310

[B74] Zhu Z. Liu Q. Jiang X. Manandhar U. Luo Z. Zheng X. (2020). The psychological status of people affected by the COVID-19 outbreak in China. *J. Psychiatr. Res.* 129 1–7. 10.1016/j.jpsychires.2020.05.026 32526513 PMC7255091

[B75] Zhu Z. Tang Y. Zhuang J. Liu Y. Wu X. Cai Y. (2019). Physical activity, screen viewing time, and overweight/obesity among Chinese children and adolescents: an update from the 2017 physical activity and fitness in China “the youth study. *BMC Public Health* 19:197. 10.1186/s12889-019-6515-9 30767780 PMC6376726

